# Muir-Torre Syndrome: A Case Report and a Literature Review of Genetic Insights and Cancer Surveillance

**DOI:** 10.7759/cureus.65828

**Published:** 2024-07-31

**Authors:** Shubam Trehan, Gurjot Singh, Kanishka Goswami, Amna Farooq, FNU Kalpana, Priya Antil, Waqas Azhar

**Affiliations:** 1 Internal Medicine, Southern Illinois University School of Medicine, Springfield, USA; 2 Internal Medicine, Memorial Medical Center, Springfield, USA; 3 Internal Medicine, St. John's Hospital, Springfield, USA; 4 Hospital Medicine, Springfield Clinic, Springfield, USA

**Keywords:** cancer surveillance, visceral neoplasms, msh6, msh2, actinic lesions, sebaceous carcinoma in situ, muir torre syndrome

## Abstract

Muir-Torre syndrome (MTS) is a rare autosomal dominant genetic disorder that manifests through the co-occurrence of sebaceous skin tumors and internal malignancies, primarily due to mutations in mismatch repair (MMR) genes such as MSH2, MLH1, and MSH6. This paper presents a detailed case report of a 57-year-old female diagnosed with MTS, highlighting her extensive medical history and the critical role of genetic testing and multidisciplinary management. The patient’s dermatological and oncological assessments revealed multiple sebaceous carcinomas and recurrent urothelial carcinoma, confirmed by a pathogenic MSH2 mutation. Through comprehensive preventive surgeries and rigorous follow-up, this case underscores the necessity of proactive cancer surveillance. The discussion integrates findings from key genetic studies and emphasizes the importance of immunohistochemistry in diagnosis. Recommendations for clinical practice include routine genetic testing, stringent surveillance, and multidisciplinary management, underscoring the need for ongoing research to understand better and manage this complex syndrome.

## Introduction

Genetic syndromes play an important role in predisposing individuals to various cancers, often providing critical insights for early detection and preventive strategies. Among these syndromes, Muir-Torre syndrome (MTS) stands out due to its distinctive manifestation of both cutaneous and internal malignancies, necessitating a comprehensive understanding by both dermatologists and oncologists. Understanding MTS is vital, as its dermatological manifestations often serve as harbingers of underlying systemic malignancies.

MTS, first described independently by Torre in 1968, is defined by the presence of sebaceous skin tumors and internal malignancies, linked primarily to germline mutations in mismatch repair (MMR) genes [1]. These early descriptions marked significant milestones in recognizing the association between dermatological and systemic malignancies. Early case studies laid the groundwork for the current understanding of MTS, emphasizing the importance of recognizing dermatological signs as potential indicators of more serious internal conditions. Recent studies have reaffirmed that mutations in MMR genes such as MLH1, MSH2, MSH3, MSH6, and PMS2 are central to the pathogenesis of MTS, with MSH2 mutations being the most prevalent [2-4]. The DNA MMR system is crucial for correcting errors that occur during DNA replication, and its impairment leads to microsatellite instability (MSI), a hallmark of both cutaneous and visceral tumors in MTS [5]. MSI results from impaired DNA MMR systems, significantly increasing mutation rates and cancer risk. This genetic mechanism underpins the high frequency of tumors observed in MTS patients.

Epidemiologically, MTS is rare, representing about 3% of colorectal and endometrial carcinoma cases and 9% of Lynch syndrome cases [6]. The syndrome typically presents a male predominance with a median diagnosis age of 53 years, encompassing a broad spectrum of malignancies, including colorectal, endometrial, ovarian, and urothelial carcinomas [7]. Additionally, other cancers such as those of the stomach, small intestine, breast, and hepatobiliary tract have been reported [8]. This wide clinical spectrum necessitates a comprehensive and vigilant approach to patient care.

Dermatological assessments are crucial, as sebaceous adenomas, carcinomas, and keratoacanthomas often precede internal malignancies. Recognizing these skin tumors should prompt consideration of MTS and further genetic evaluation. Case examples underscore the importance of early dermatological assessment in leading to timely diagnosis and intervention for internal malignancies [8]. The role of genetic testing in diagnosing MTS cannot be overstated. Comprehensive gene panels and next-generation sequencing (NGS) have improved the detection rate of pathogenic variants, aiding in the diagnosis and risk stratification of affected individuals and their families [3]. The integration of clinical, histopathological, and molecular data is essential for accurate diagnosis and risk assessment. Genetic testing for germline mutations in MMR genes is recommended for patients with clinical features suggestive of MTS.

Given the increased risk of multiple malignancies, stringent surveillance protocols are essential for individuals with MTS. Regular dermatological examinations, colonoscopies, endoscopic evaluations, and imaging studies are recommended to detect new or recurrent malignancies at an early stage [7]. Prophylactic surgeries, such as colectomy or hysterectomy, may be considered in high-risk individuals to reduce the cancer burden. Multidisciplinary management involving dermatologists, oncologists, gastroenterologists, geneticists, and surgeons is crucial for the comprehensive care of MTS patients. Advances in genetic testing and molecular diagnostics hold promise for the early detection and personalized management of MTS. Ongoing research is focused on identifying novel genetic variants and elucidating the molecular mechanisms underlying MTS, which may lead to targeted therapies and improved surveillance strategies [4]. Furthermore, exploring the psychosocial impact of living with a hereditary cancer syndrome and its effect on quality of life can inform supportive care strategies to better support these patients and their families.

Emerging data suggest that immunotherapy may have a role in the management of MMR-deficient tumors, including those associated with MTS. Clinical trials evaluating the efficacy of immune checkpoint inhibitors in MMR-deficient cancers are ongoing, and preliminary results are promising [3]. The integration of immunotherapy into the treatment paradigm for MTS could potentially improve outcomes for patients with advanced or recurrent malignancies. This case report aims to provide a comprehensive review of MTS, backed by extensive research evidence, to enhance the understanding and management of this complex syndrome. Through a detailed examination of a 57-year-old female diagnosed with MTS, the report will highlight the critical role of genetic testing, multidisciplinary management, and the importance of proactive cancer surveillance.

## Case presentation

**Figure 1:**
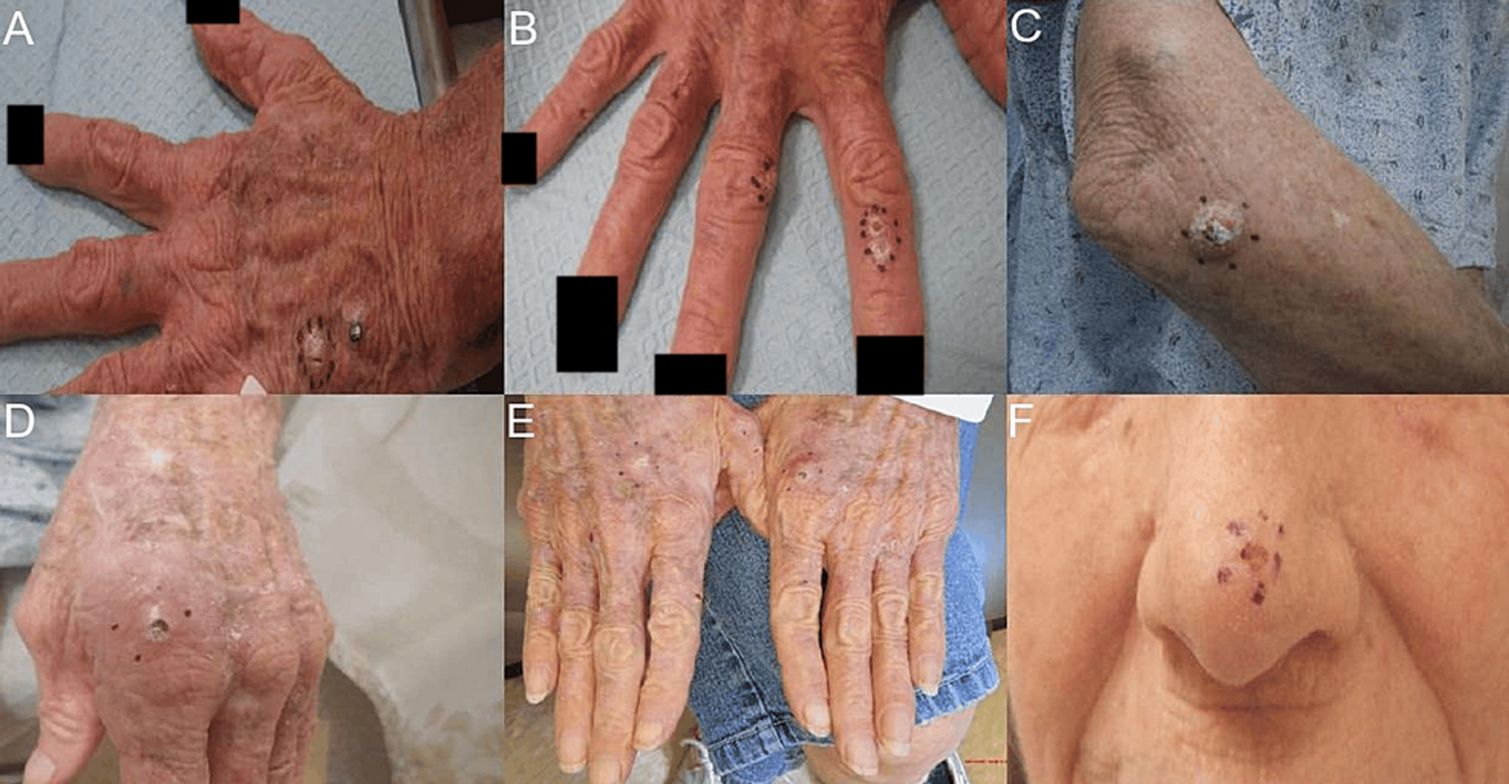
Lesions at different parts of the body Images A-E show erythematous scaling papules on the hands and right arm. Image F shows an actinic macule over the nose. Image Credits: Waqas Azhar

A 57-year-old female with a complex medical history including hypertension, hyperlipidemia, hypothyroidism, carotid artery stenosis, peripheral artery disease, rheumatoid arthritis, Barrett’s esophagus, and emphysema presented for dermatological evaluation. Her initial assessment noted diffuse actinic changes, erythematous scaling macules, and papules on her hands, arms, and face (Figure 1). These actinic lesions were treated with liquid nitrogen.

**Figure 2:**
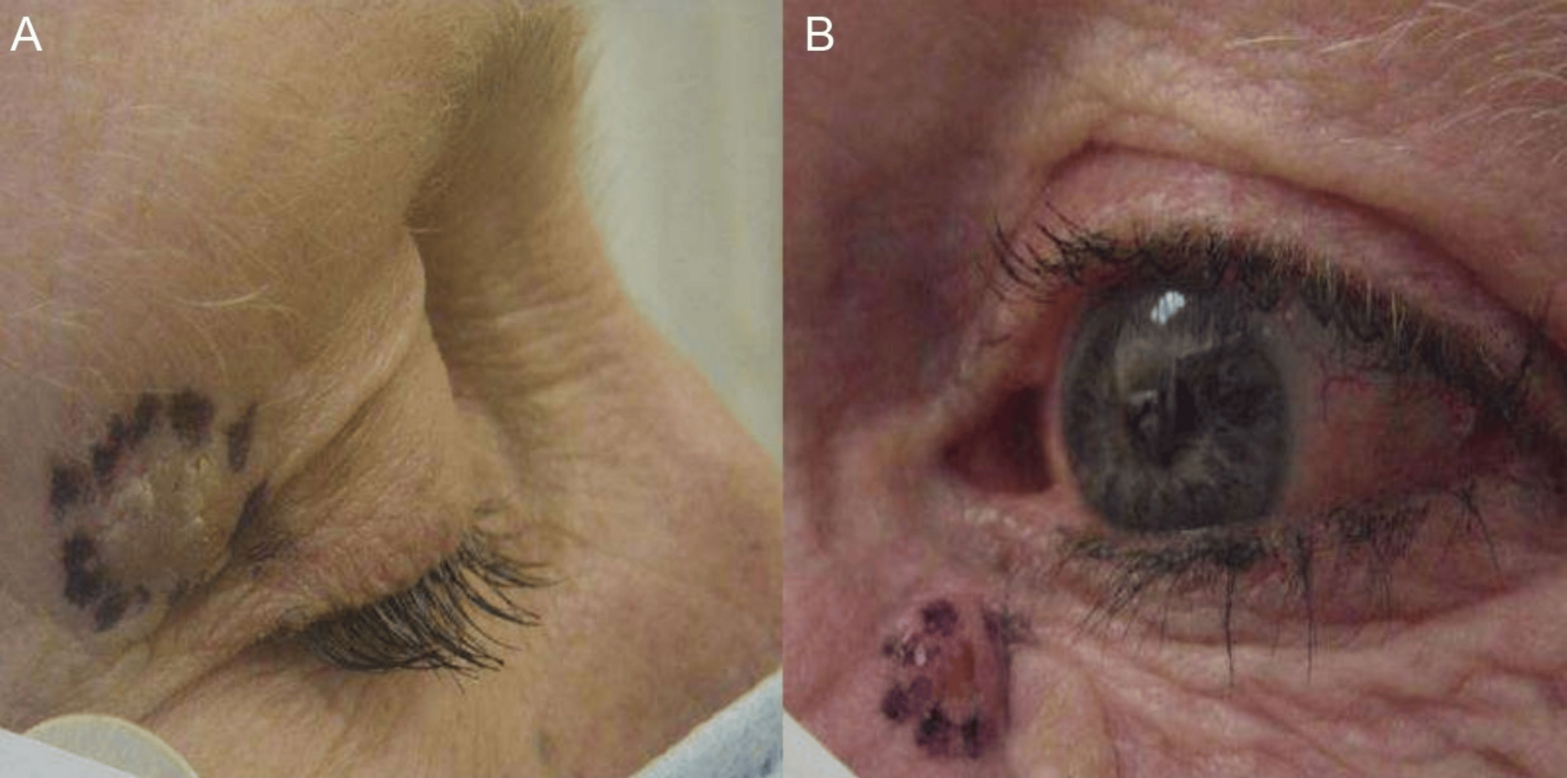
Lesion around the eyes Image A shows a yellowish translucent umbilicated papule near the right upper eyelid. Image B shows a pink-crusted papule near the left lower eyelid. Image Credits: Waqas Azhar

One year later, during a follow-up visit, the patient presented with a new pink-crusted papule near the left lower eyelid and a yellowish translucent umbilicated papule near the right upper eyelid (Figure 2).

**Figure 3:**
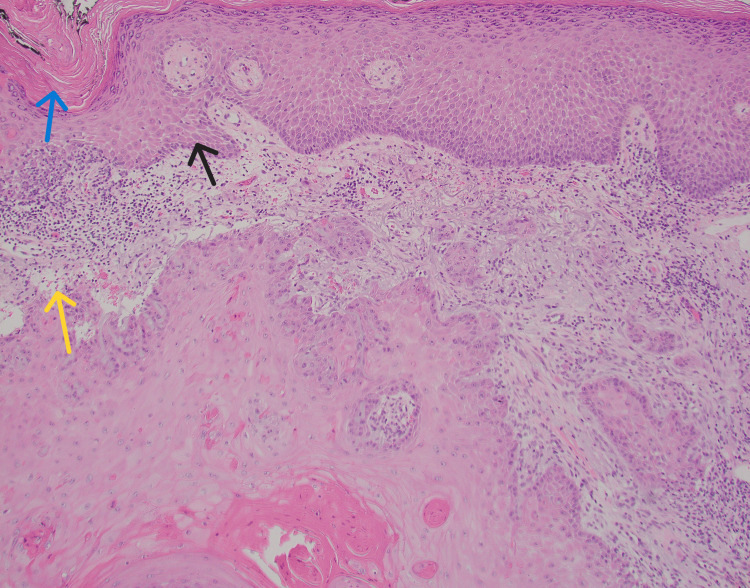
Biopsy showing features of squamous cell carcinoma which are cellular atypia (blue arrow), keratinization (black arrow), and yellow arrow showing vacuolization within the cytoplasm of the tumor cells. Image Credit: Waqas Azhar

Both lesions were excised using Mohs micrographic surgery, and biopsies were sent for histopathological evaluation. The biopsy results received a week later indicated features consistent with sebaceous carcinoma in situ (Figure 3).

**Figure 4:**
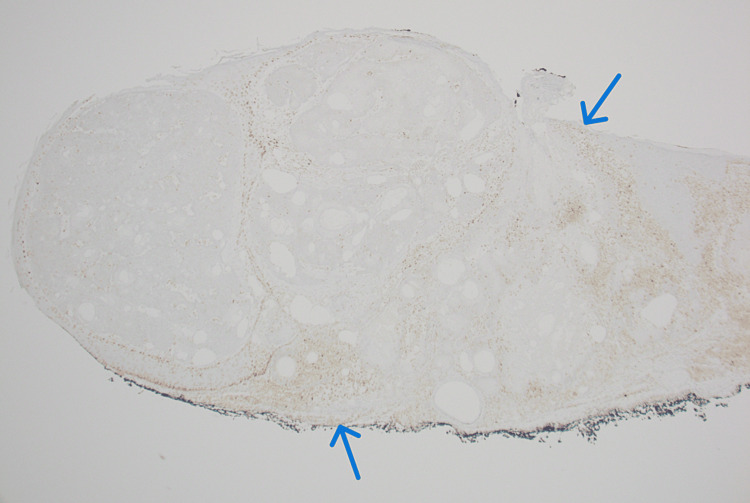
Biopsies showing sebaceous carcinoma in situ with immunohistochemical loss of staining for MSH-2 Image Credits: Waqas Azhar

**Figure 5:**
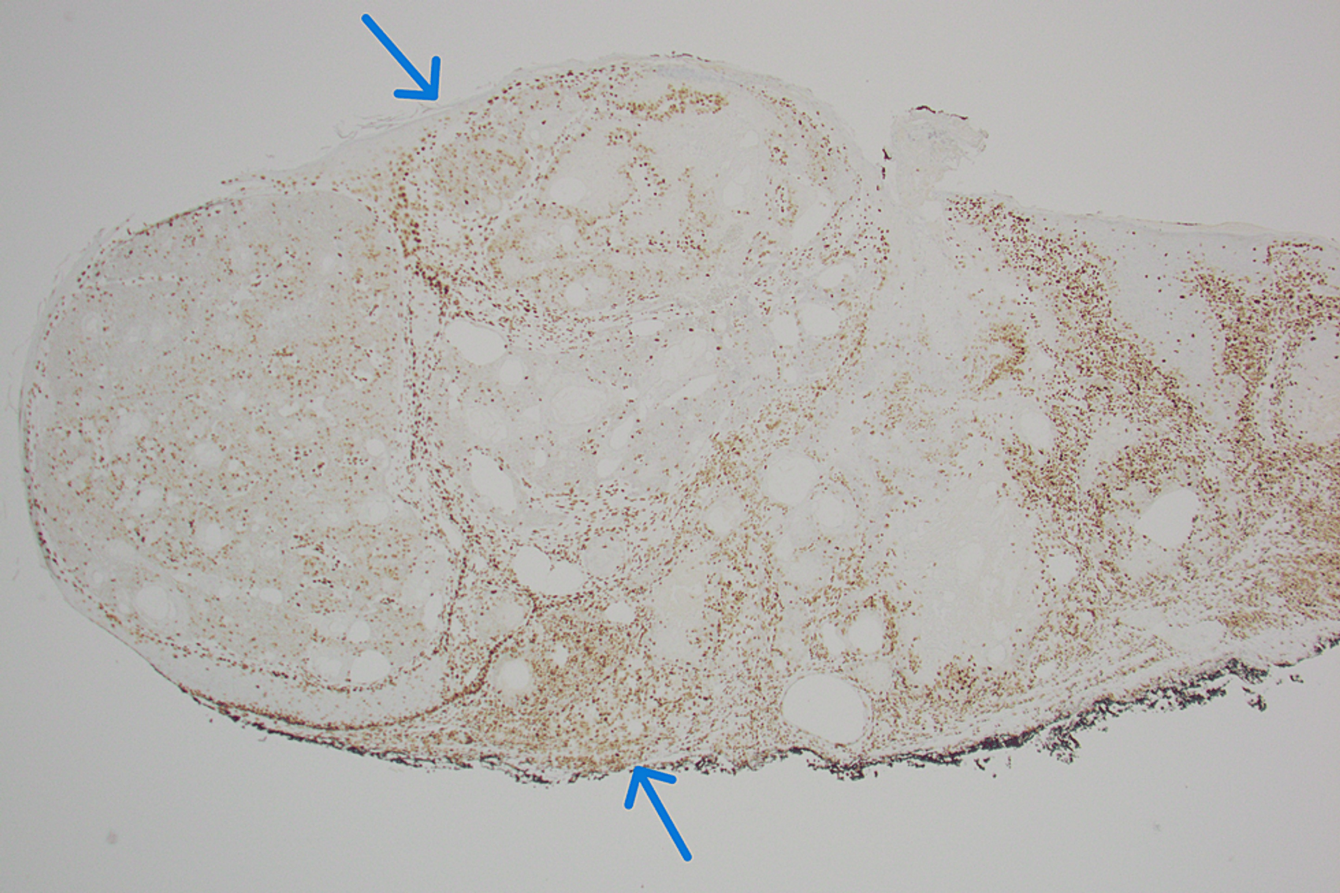
Biopsies showing sebaceous carcinoma in situ with immunohistochemical loss of staining for MSH-6 Image Credits: Waqas Azhar

Further testing with immunohistochemistry confirmed the diagnosis of sebaceous carcinoma in situ, showing a loss of staining for MSH-2 and MSH-6 (Figures 4-5). The diagnosis of sebaceous carcinoma in situ raised the suspicion for MTS, given its frequent association with the syndrome.

A detailed review of the patient's history revealed significant gynecological and gastrointestinal concerns. She had a history of HPV-positive atypical squamous cells and low-grade squamous intraepithelial lesions treated in her 30s, and she underwent uterine interventions for abnormal bleeding in her mid-40s. Her initial screening colonoscopies revealed hyperplastic and adenomatous polyps, suggesting a genetic predisposition to malignancy.

More recently, the patient developed hematuria, leading to a CT scan that identified a 3.5 cm lesion in the renal pelvis. Subsequent cystoscopy and biopsy diagnosed low-grade and focal high-grade non-invasive papillary urothelial carcinoma. Given her risk profile, she elected to undergo a prophylactic hysterectomy along with a nephron-ureterectomy for the renal lesion. Surgical pathology confirmed high-grade non-invasive papillary urothelial carcinoma in the renal pelvis. Follow-ups have shown recurrent urothelial lesions, treated with electrofulguration and mitomycin.

The presence of sebaceous carcinoma in situ, in conjunction with multiple neoplasms involving visceral organs, confirms the diagnosis of MTS in this patient.

## Discussion

MTS is a rare autosomal dominant condition representing a clinical variant of Lynch syndrome. The syndrome is characterized by the co-occurrence of sebaceous skin tumors and internal malignancies, primarily due to germline mutations in DNA MMR genes. These mutations result in MSI, which is a hallmark of the syndrome and leads to a high mutation rate across the genome [9,10].

Genetics and pathophysiology

The genetic basis of MTS lies predominantly in mutations affecting MMR genes, including MLH1, MSH2, MSH6, PMS2, and occasionally EPCAM deletions that inactivate MSH2. Among these, MSH2 mutations are most commonly associated with MTS, accounting for a significant proportion of cases [3,4]. The MMR system is essential for correcting DNA replication errors, and its dysfunction leads to MSI, observed in both cutaneous and internal tumors [5]. Studies have demonstrated that the loss of MSH2 and MSH6 protein expression in tumors can serve as a diagnostic marker for MTS, facilitating early detection and management (Table 1) [11,12].

**Table 1:** Key genetic mutations in Muir-Torre syndrome (MTS)

Gene	Mutation Type	Frequency in MTS	Function	Reference
MSH2	Germline	High	DNA mismatch repair	[9]
MLH1	Germline	Moderate	DNA mismatch repair	[10]
MSH6	Germline	Low	DNA mismatch repair	[8]
PMS2	Germline	Rare	DNA mismatch repair	[5]
EPCAM	Deletion	Rare	MSH2 inactivation via EPCAM deletion	[3]

Clinical features and epidemiology

MTS is an infrequent disorder, constituting approximately 3% of all colorectal and endometrial carcinomas and around 9% of Lynch syndrome cases [6]. The syndrome shows a male predominance with a male-to-female ratio of 3:2, and the median age of diagnosis is 53 years [7]. The clinical spectrum of MTS includes a variety of visceral malignancies, with colorectal carcinoma being the most common, followed by endometrial, ovarian, and urothelial carcinomas. Additionally, other cancers such as those of the stomach, small intestine, breast, and hepatobiliary tract have been reported (Table 2) [8,13].

**Table 2:** Other diseases associated with Muir-Torre syndrome (MTS) MSI: microsatellite instability; MMR: mismatch repair

Disease	Associated Gene	Mechanism of Association	Frequency in MTS	Reference
Colorectal carcinoma	MSH2, MLH1	MSI and loss of MMR	High	[13]
Endometrial carcinoma	MSH2, MLH1	MSI and loss of MMR	Moderate	[3]
Ovarian carcinoma	MSH2	MSI and loss of MMR	Low	[4]
Urothelial carcinoma	MSH2	MSI and loss of MMR	Moderate	[4]
Small intestine carcinoma	MSH2	MSI and loss of MMR	Rare	[8]
Gastric carcinoma	MSH2, MLH1	MSI and loss of MMR	Rare	[5]
Breast carcinoma	MSH2, MLH1	MSI and loss of MMR	Low	[3]
Sebaceous neoplasms	MSH2, MLH1	MSI and loss of MMR	High	[4]
Hepatobiliary carcinoma	MSH2, MLH1	MSI and loss of MMR	Rare	[5]
Pancreatic carcinoma	MSH2, MLH1	MSI and loss of MMR	Rare	[3]
Brain tumors (Turcot syndrome)	APC, MLH1, MSH2	MSI and loss of MMR	Very rare	[6]

Diagnostic strategies

The diagnosis of MTS is primarily clinical, supported by histopathological and immunohistochemical findings. Immunohistochemical loss of MMR protein expression in tumors, particularly MSH2 and MSH6, is a critical diagnostic feature [4]. Genetic testing for germline mutations in MMR genes is recommended for patients with clinical features suggestive of MTS. Various genetic testing options, including comprehensive gene panels, NGS, and multiplex ligation-dependent probe amplification (MLPA), enhance the detection of pathogenic variants. For instance, a study highlights the role of immunohistochemistry and genetic testing in diagnosing a complex case involving concurrent sebaceous carcinoma and colon cancer, stressing the necessity of advanced diagnostic tools in managing MTS effectively [14,15].

Management and surveillance

Given the increased risk of multiple malignancies, stringent surveillance protocols are essential for individuals with MTS. Regular dermatological examinations, colonoscopies, endoscopic evaluations, and imaging studies are recommended to detect new or recurrent malignancies at an early stage [16]. Prophylactic surgeries, such as colectomy or hysterectomy, may be considered in high-risk individuals to reduce the cancer burden [6]. Multidisciplinary management involving dermatologists, oncologists, gastroenterologists, geneticists, and surgeons is crucial for the comprehensive care of MTS patients. Regular multidisciplinary meetings can facilitate the development of individualized surveillance and management plans, ensuring optimal outcomes [8]. In addition to regular surveillance, specific treatments for MTS-related lesions include surgical excision of sebaceous adenomas and carcinomas, as well as cryotherapy for keratoacanthomas. Emerging therapies such as immunotherapy, particularly checkpoint inhibitors like pembrolizumab and nivolumab, have shown promise in treating MMR-deficient tumors and offer new hope for patients with advanced or recurrent malignancies (Table 3) [11,12].

**Table 3:** Surveillance protocol for Muir-Torre syndrome

Cancer Type	Surveillance Method	Frequency	Starting Age	Procedure	References
Dermatological Surveillance					
Sebaceous Neoplasms and Keratoacanthomas	Full-body skin examination	Every 6-12 months	Late teens/early twenties	Dermatological examination with dermoscopy. Biopsy any suspicious lesions.	[3,4]
Gastrointestinal Surveillance					
Colorectal Carcinoma	Colonoscopy	Every 1-2 years	Age 20-25 or earlier if family history	High-resolution colonoscopy with chromoendoscopy or narrow-band imaging. Polypectomy for any detected polyps.	[6]
Small Intestine Carcinoma	Capsule endoscopy/small bowel enteroscopy	Every 2-3 years	Age 20-25 or earlier if family history	Capsule endoscopy for non-invasive screening. Follow-up with enteroscopy if abnormalities are detected.	[8]
Gynecological Surveillance					
Endometrial Carcinoma	Transvaginal ultrasound, endometrial biopsy	Annually	Age 30-35	Transvaginal ultrasound to measure endometrial thickness. Biopsy if abnormalities are detected.	[7]
Ovarian Carcinoma	Transvaginal ultrasound, serum CA-125	Annually	Age 30-35	Transvaginal ultrasound and CA-125 blood test as a tumor marker.	[7,9]
Urological Surveillance					
Urothelial Carcinoma	Urine cytology, cystoscopy	Annually	Age 30-35	Urine cytology to detect abnormal cells. Cystoscopy to visualize the bladder and urethra for any lesions.	[10]
Upper Gastrointestinal Surveillance					
Gastric Carcinoma	Esophagogastroduodenoscopy (EGD)	Every 2-3 years	Age 30-35	EGD to inspect the esophagus, stomach, and duodenum for abnormalities. Biopsy of suspicious lesions.	[5]
Hepatobiliary and Pancreatic Surveillance					
Hepatobiliary and Pancreatic Carcinomas	MRI/MRCP or ultrasound	Every 1-2 years	Age 30-35	MRI with magnetic resonance cholangiopancreatography (MRCP) or abdominal ultrasound to detect lesions in the liver, bile ducts, and pancreas.	[5]
Breast Cancer Surveillance					
Breast Carcinoma	Mammography, breast MRI	Annually	Age 30-35	Mammography for early detection of breast cancer, supplemented by MRI for higher sensitivity in dense breast tissue.	[3]
Psychological and Genetic Counseling					
Psychosocial Support	Psychological support and counseling	As needed	Ongoing	Regular psychological support and counseling to help patients cope with anxiety and stress associated with high cancer risk.	[3]
Genetic Counseling	Genetic counseling	As needed	Ongoing	Ongoing genetic counseling to inform patients and their families about their risks, surveillance strategies, and reproductive options.	[3]
Prophylactic Surgery					
Prophylactic Hysterectomy and Bilateral Salpingo-Oophorectomy	Surgical intervention	As recommended by healthcare provider	After childbearing is complete or at age 40	Considered to reduce the risk of gynecological cancers in high-risk individuals.	[7]

Prognosis

The prognosis of MTS is influenced by several factors:

Type and Stage of Malignancies

Early detection and treatment of cutaneous tumors and internal malignancies significantly improve outcomes. For instance, early-stage colorectal cancer detected through regular surveillance can be managed effectively, leading to a favorable prognosis [7].

Effectiveness of Surveillance and Preventive Measures

Stringent surveillance protocols and prophylactic surgeries can reduce cancer risk and improve overall prognosis [6].

Genetic Factors

Specific MMR gene mutations (e.g., MSH2) influence the risk profile and prognosis. Patients with MSH2 mutations may require more rigorous surveillance due to a higher predisposition to various cancers [3].

Treatment Options

Standard treatments (surgery, chemotherapy, radiation therapy) and emerging therapies (immunotherapy) play crucial roles in determining patient outcomes. For example, pembrolizumab has shown efficacy in treating MMR-deficient colorectal cancer, potentially improving the prognosis for MTS patients [11].

Multidisciplinary Management

Comprehensive care involving multiple specialties ensures all aspects of MTS are addressed, leading to better patient outcomes [8].

Psychosocial Factors

Psychological support and counseling improve the quality of life and overall prognosis by helping patients cope with the anxiety and stress associated with the risk of multiple cancers [3].

Recent advances and future directions

Recent studies have also explored the molecular landscape of MTS, identified novel pathogenic variants, and expanded the genotype-phenotype correlations. For example, a study reviewed the emerging molecular techniques for diagnosing MTS and other Lynch syndrome variants, highlighting the potential of NGS in clinical practice [3]. Abbas and Mahalingam (2009) described a rare frame-shift mutation in MSH2, contributing to the expanding mutational spectrum of MTS [5]. Insights from novel sequencing technologies such as spatial transcriptomics could inform resistance mechanisms and recurrence in MTS-related cancers, potentially guiding more effective treatment strategies [17]. These findings underscore the importance of continuous advancements in genetic testing for better diagnosis and management.

Advances in genetic testing and molecular diagnostics hold promise for the early detection and personalized management of MTS. Ongoing research is focused on identifying novel genetic variants and elucidating the molecular mechanisms underlying MTS, which may lead to targeted therapies and improved surveillance strategies [4]. Furthermore, exploring the psychosocial impact of living with a hereditary cancer syndrome and the effect on quality of life can inform supportive care strategies to better support these patients and their families [3]. Emerging data suggest that immunotherapy may have a role in the management of MMR-deficient tumors, including those associated with MTS. Clinical trials evaluating the efficacy of immune checkpoint inhibitors in MMR-deficient cancers are ongoing, and preliminary results are promising [11,12]. The exploration of new therapeutic targets such as VISTA (V-domain Ig suppressor of T cell activation) may provide innovative treatment options for patients with MMR-deficient tumors, expanding the current therapeutic landscape [18]. The integration of immunotherapy into the treatment paradigm for MTS could potentially improve outcomes for patients with advanced or recurrent malignancies.

## Conclusions

MTS is a complex autosomal dominant condition marked by the coexistence of sebaceous skin tumors and internal malignancies due to germline mutations in MMR genes. Genetic testing, particularly NGS, plays an important role in identifying pathogenic variants, enabling precise risk stratification and personalized surveillance strategies. These measures facilitate early detection and intervention, significantly improving patient outcomes. The integration of dermatological and oncological assessments, preventive surgeries, and rigorous follow-up protocols is crucial in managing MTS. Additionally, the emerging role of immunotherapy offers promising new treatment options for MMR-deficient tumors, potentially improving the prognosis for patients with advanced or recurrent malignancies. Advancements in genetic testing and molecular diagnostics, coupled with multidisciplinary collaboration, are essential for the effective management of MTS. Future research should focus on uncovering the molecular mechanisms of MTS, identifying novel genetic variants, and developing biomarkers to improve the management and prognosis of MTS.
